# Case report: Metastatic melanoma masquerading as apical hypertrophic cardiomyopathy

**DOI:** 10.3389/fcvm.2022.993631

**Published:** 2022-12-09

**Authors:** Muddasir Ashraf, Arshad Jahangir, M. Fuad Jan, Lakshmi Muthukumar, Gary Neitzel, A. Jamil Tajik

**Affiliations:** ^1^Aurora Cardiovascular and Thoracic Services, Aurora Sinai/Aurora St. Luke’s Medical Centers, Advocate Aurora Health, Milwaukee, WI, United States; ^2^Department of Pathology and Laboratory Medicine, Aurora St. Luke’s Medical Center, Advocate Aurora Health, Milwaukee, WI, United States

**Keywords:** left ventricular apex, metastatic melanoma, tumor, case report, apical hypertrophic cardiomyopathy

## Abstract

**Background:**

Cardiac tumors are usually metastatic. Melanoma is the tumor with the highest rate of cardiac metastasis. Clinicians need to be aware of the metastatic involvement of the left ventricular apex as a differential diagnosis of apical hypertrophic cardiomyopathy.

**Case summary:**

A 74-year-old woman presented for evaluation of fatigue. The initial electrocardiogram and echocardiogram showed features of apical hypertrophic cardiomyopathy. The patient reported a lesion on her right forearm that had been present for many years, leading to its biopsy, which showed melanoma. Further evaluation with a chest-computed tomography (CT) scan showed left lung nodules and nodular thickening of the left ventricular apex. Positron emission tomography showed an increased uptake of fluorodeoxyglucose in the left lung nodule and left ventricular apex, suggestive of metastatic spread of the melanoma. A CT-guided biopsy of the left lung nodule revealed melanoma. The patient was treated with ipilimumab initially, followed by paclitaxel with poor response to treatment, and later passed under hospice care.

**Conclusion:**

Metastatic tumors involving the left ventricular apex should be considered in the differential diagnosis of apical hypertrophic cardiomyopathy, especially in patients with a history of melanoma, and advanced cardiac imaging, including cardiac magnetic resonance imaging, CT, and/or positron emission tomography (PET) may help with narrowing down the differential diagnosis.

## Introduction

Cardiac tumors are usually metastatic, with a reported incidence 100 times higher than that of primary cardiac tumors. The reported incidence of cardiac metastasis is 0.7–3.5% in the general population, with rates up to 9.1% in patients diagnosed with metastatic cancer. Metastatic melanoma has a higher rate of metastasis to the heart than any other tumor (1). Metastatic melanoma should always be considered a differential diagnosis of apical hypertrophy cardiomyopathy, especially in patients with a history of melanoma. We present a 74-year-old woman who was initially diagnosed with apical hypertrophic cardiomyopathy, but further evaluation and diagnostic testing revealed metastatic involvement of the left ventricular apex due to melanoma.

## Case summary

**FIGURE 1:**
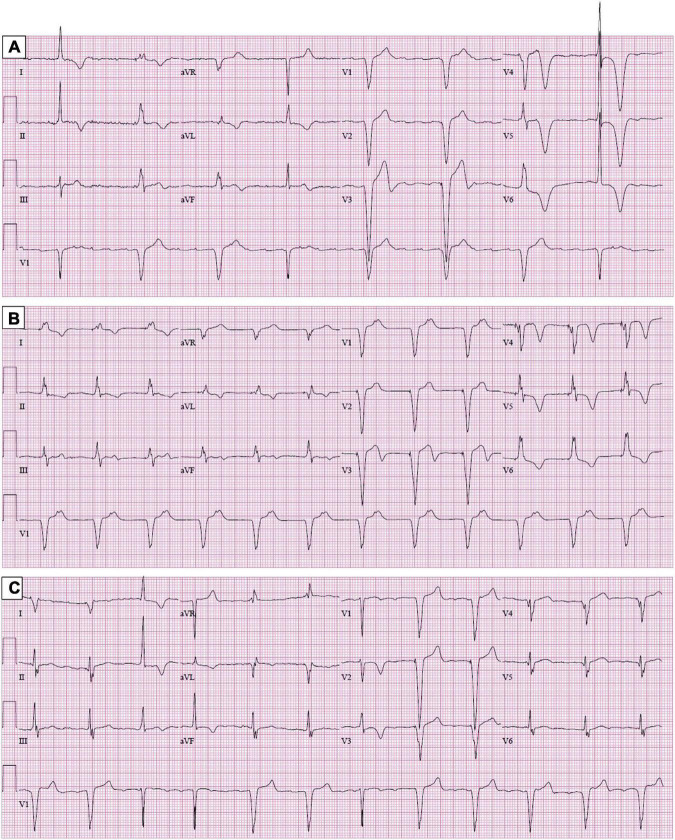
Electrocardiogram. Panel **(A)** shows tall R waves and giant-negative T waves in V4–V6. Panels **(B)** (8 months later) and **(C)** (24 months later) show a progressive decrease in the amplitude of the R waves and T wave inversions in the paced beats in precordial leads.

A 74-year-old female, who had a past medical history of chronic atrial fibrillation on daily warfarin, moderate pulmonary hypertension, and a pacemaker placement 4 years prior for complete heart block, came in for an evaluation of fatigue (Table 1). The initial 12-lead electrocardiogram showed demand ventricular pacing with underlying atrial fibrillation, left ventricular (LV) hypertrophy, and giant negative T waves in paced as well as normally conducted beats (Figure 1A), and a transthoracic echocardiogram (TTE) showed apical hypertrophy. Physical examination showed variable-intensity S1, normal intensity S2, point of maximal impulse non-displaced, grade I/VI systolic murmur along the left sternal border and apex, and an S3. A plaque-like 2.5 × 2 cm lesion was observed on the right forearm, which the patient said had been present for many years.

**TABLE 1:** Timeline of events.

Initial visit	The patient presented with fatigue and was diagnosed with apical hypertrophic cardiomyopathy based on an initial electrocardiogram and transthoracic echocardiogram.
One month later	The patient underwent punch biopsy of the right forearm lesion, which was positive for melanoma.
1.5 months later	Wide excision of the right forearm lesion and sentinel lymph node biopsy of right axilla done, and patient found to have Stage IIC melanoma.
20 months later	A computed tomography (CT) scan of the chest and a whole-body positron emission tomography (PET) scan were suggestive of metastatic spread of melanoma to the left ventricular apex and left lung. A CT-guided biopsy of the left pulmonary nodule confirmed metastatic melanoma.
21 months later	The patient was started on ipilimumab as she was negative for *BRAF V600* mutation.
23 months later	Ipilimumab was stopped due to a significant increase in liver enzymes.
24 months later	Patient started on paclitaxel.
32 months later	Repeat PET scan showed progressive disease. Patient started on hospice care and passed at a hospice facility.

Differential diagnoses of apical wall thickening and T wave inversion in precordial leads considered are shown in Supplementary Tables 1, 2, respectively.

**FIGURE 2:**
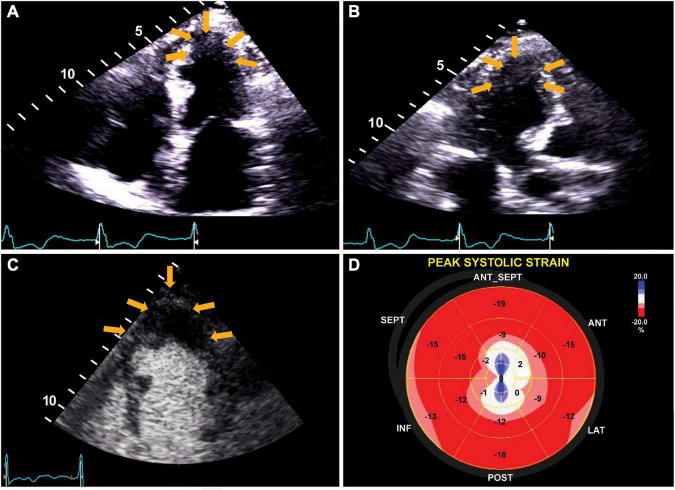
Transthoracic echocardiogram. The apical four-chamber view **(A)** (Supplementary Video 1), apical long-axis view **(B)** (Supplementary Video 2), and definity contrast echocardiogram **(C)** (Supplementary Video 3) show apical wall thickness (yellow arrows). Panel **(D)** shows strain analysis with markedly reduced peak systolic strain and evidence of dyskinesis in the apical segments.

**FIGURE 3:**
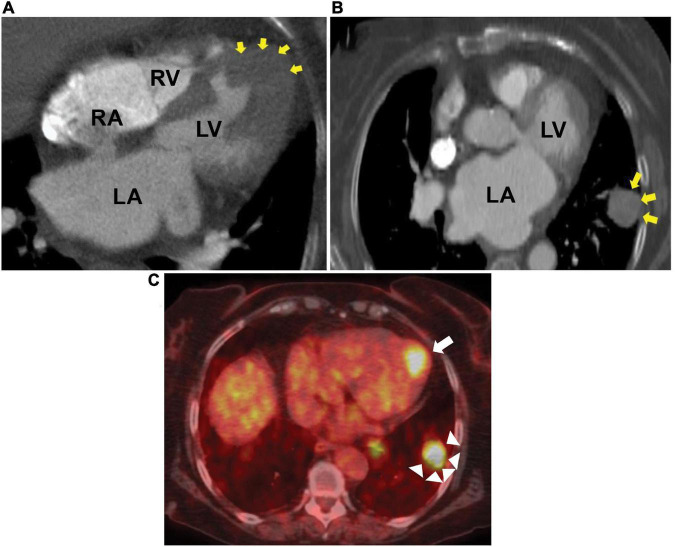
Chest CT and PET scan. Computed tomography (CT) showing metastatic melanoma involving the left ventricular apical wall **(A)**, left lung **(B)**, and positron emission tomography (PET) scan axial image **(C)** showing increased fluorodeoxyglucose uptake in the left ventricular apex (arrow) and left lung (arrow heads). LA, left atrium; LV, left ventricle; RA, right atrium; RV, right ventricle.

Laboratory workup, including complete blood count, complete metabolic panel, pro-B-type natriuretic peptide, and ultrasensitive troponin, was unremarkable. Chest X-ray was normal. The subsequent 12-lead electrocardiograms showed a decreasing amplitude of T wave inversions and R waves over time in the paced beats (Figures 1B,C). A TTE showed an apical wall thickness of 19 mm (Figure 2; Supplementary Videos 1–3). The global longitudinal strain was reduced to −9% and the LV ejection fraction was 70%. A punch biopsy of the right forearm lesion showed nodular, amelanotic melanoma with Breslow tumor thickness of at least 2.2 mm and at least Clark level IV invasion. Subsequently, this was resected and considered T4B N0 M0, stage II-C melanoma. The chest computed tomography (CT) s162can showed nodular thickening of the LV apex and pulmonary nodules in the left lung, suggestive of metastatic disease (Figures 3A,B). A positron emission tomography (PET) scan revealed increased uptake of fluorodeoxyglucose (FDG) in the LV apex and left lung nodules (Figure 3C), suggestive of metastatic spread of the melanoma to the LV apex and left lung. CT-guided biopsy of the pulmonary nodule confirmed the metastatic spread of the melanoma to the lungs (see Supplementary Figure 1). Hematoxylin and eosin staining revealed a diffuse sheet of malignant epithelioid cells with moderately pleomorphic, hyperchromatic nuclei, and poorly defined cytoplasm without pigments. The tumor also expressed melanoma markers S100 and MART-1.

The patient was initially started on metoprolol succinate 25 mg daily for apical hypertrophic cardiomyopathy (ApHCM). Once the diagnosis of metastatic melanoma was confirmed, she was started on ipilimumab, a cytotoxic T-lymphocyte-associated protein 4 inhibitor. However, the patient’s liver enzymes increased to greater than five times the normal, thus it was discontinued. PD-1 inhibitors were not approved by the Food and Drug Administration for metastatic melanoma at that time. The patient was subsequently started on paclitaxel, but the disease progressed, and the patient felt weaker with no significant improvement in her symptoms. The patient was actively involved in the decision-making. She did not want any further treatment and was placed in hospice care, where she expired.

## Discussion

In an autopsy study of patients with metastatic melanoma, 64% had cardiac metastases (2). The metastatic spread is usually through hematogenous dissemination (1). It usually involves the right atrium (3, 4). Apical involvement is rare (5, 6). Cardiac metastasis could be missed unless specific symptoms are present or specific imaging is performed.

Asymptomatic massive involvement of the myocardium in metastatic melanoma has been described as a “charcoal heart.” The most common presenting symptom in patients with cardiac melanoma is shortness of breath, but chest pain, syncope, fatigue, and weakness have also been described.

Electrocardiographic changes suggestive of the ischemic pattern, including ST depression and T wave inversion, are more common in patients with cardiac metastasis. Cates et al. analyzed electrocardiograms obtained within 3 months prior to death in patients diagnosed with cancer premortem or postmortem and found an ischemic pattern in 40% of patients with cardiac metastasis compared to <1% in patients without cardiac metastasis (7). In the present case, the patient showed tall R waves and giant negative T waves in precordial leads. To our knowledge, this is the first case of cardiac melanoma with giant negative T waves mimicking the apical variant of hypertrophic cardiomyopathy. The patient’s serial 12-lead electrocardiograms showed the resolution of giant negative T waves over 2 years. These serial changes in patients diagnosed with ApHCM may suggest additional processes, such as the progression of fibrosis, new myocardial infarction, or infiltrative disease.

A transthoracic echocardiogram is the most frequently used non-invasive test to evaluate benign and malignant cardiac lesions and as a follow-up test for response to therapy. It can diagnose cardiac melanoma in up to 80% of cases (4).

The chest CT scan can help identify metastatic lesions in both cardiac and non-cardiac sites. Cardiac magnetic resonance imaging (CMR) is usually helpful in further characterizing these lesions, but it could not be obtained in our patient owing to a pacemaker-dependent underlying complete heart block.

The PET scan helps identify increased metabolic activity in the regions affected by the tumor by measuring ^18^F-FDG uptake by the tumor. It is more than 90% sensitive and specific in diagnosing malignant cardiac tumors (8).

The treatment of metastatic melanoma depends on the presence of a *BRAF*V600 mutation. Immune checkpoint inhibitors are used as the first-line treatment in patients who are negative for the mutation. The National Comprehensive Cancer Network recommends programmed cell death 1 (PD-1) inhibitors for these patients. Patients who harbor *BRAF*V600 mutation are usually treated with targeted therapies, including BRAF and MEK inhibitors (9). Median survival is 12 months after diagnosis, with poor prognosis in chemotherapy-resistant cases (4, 10).

## Conclusion

The diagnosis of metastatic melanoma to the LV apex should be considered in patients with ApHCM, especially those with a history of melanoma. The resolution of negative T waves in the precordial leads in patients diagnosed with ApHCM should alert for additional processes, such as progression of fibrosis, new myocardial infarction, or infiltrative disease, such as cardiac metastasis involving the LV apex, as illustrated by this case. Cardiac imaging, including CMR, CT, and/or PET plays a vital role in narrowing down the differential diagnosis.

## Data availability statement

The original contributions presented in this study are included in this article/Supplementary material, further inquiries can be directed to the corresponding author.

## Ethics statement

Written informed consent was obtained from the individual(s) for the publication of any potentially identifiable images or data included in this article.

## Author contributions

All authors listed have made a substantial, direct, and intellectual contribution to the work, and approved it for publication.
