# Supplementary figures and images for: Case report: Metastatic melanoma masquerading as apical hypertrophic cardiomyopathy
Source: Front Cardiovasc Med. 2022 Dec 9;9:993631. doi: 10.3389/fcvm.2022.993631 (PMC9780589; doi:10.3389/fcvm.2022.993631)

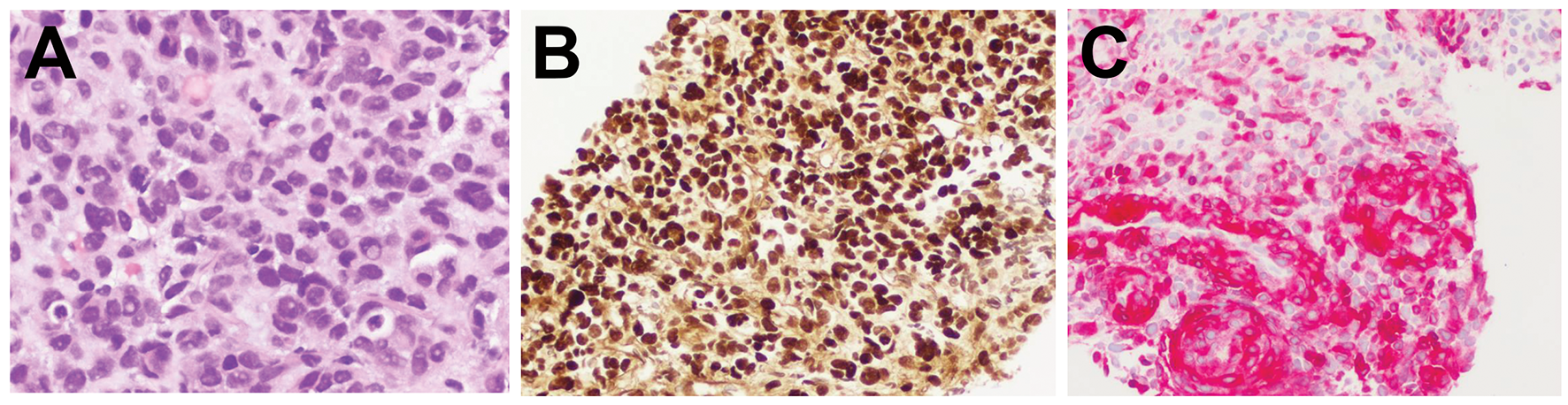

Supplement: Supplementary file 4 [file Image_1.TIF]
