# Supplementary material for: Case report: Metastatic melanoma masquerading as apical hypertrophic cardiomyopathy
Source: Front Cardiovasc Med. 2022 Dec 9;9:993631. doi: 10.3389/fcvm.2022.993631 (PMC9780589; doi:10.3389/fcvm.2022.993631)
Supplement: Supplementary file 5 [file Data_Sheet_1.docx]

**Supplemental Figure Legend**

**Supplemental Figure 1: Tissue pathology**

The hematoxylin and eosin images **(A)** reveal a diffuse sheet of malignant epithelioid cells with moderately pleomorphic, hyperchromatic nuclei and poorly defined cytoplasm without pigment. The tumor expressed the melanoma markers S100 with strong nuclear staining **(B)** and MART-1 with strong cytoplasmic staining **(C)**.

**Supplemental Tables**

**Supplemental Table 1: Differential diagnosis of apical wall thickening**

| **Differential diagnosis of apical wall thickening** |
| --- |
| Apical hypertrophy |
| Apical left ventricular noncompaction |
| Infiltrative disease |
| Hypereosinophilic syndrome with endomyocardial fibrosis |
| Metastatic disease involving the left ventricular apex |

**Supplemental Table 2: Differential diagnosis of T wave inversion in the precordial leads**

| **Differential diagnosis of T wave inversion in the precordial leads** |
| --- |
| Apical variant of hypertrophic cardiomyopathy |
| Arrhythmogenic ventricular cardiomyopathy |
| Acute coronary syndrome |
| Pericarditis |
| Myocarditis |
| Acute central nervous system disorders (subarachnoid hemorrhage, electroconvulsive therapy) |
| Acute adrenergic stress |
| Takotsubo cardiomyopathy |
| Left ventricular noncompaction |
| Pheochromocytoma |
| Acute right ventricular overload |
| Pulmonary edema |
| Pulmonary embolism |
| Cardiac memory T-wave secondary to transient tachycardia or post-ventricular pacing states |
| Athlete’s heart |
| Antiarrhythmic drug effects or cocaine use |
| Sarcoidosis |
| Infiltrative myocardial disease including metastatic tumors |
|  |

**VIDEO LEGENDS**

**Supplemental Video 1:**

Transthoracic echocardiogram apical 4-chamber view showing apical wall thickening.

**Supplemental Video 2:**

Transthoracic echocardiogram apical long axis-view view showing apical wall thickening.

**Supplemental Video 3:**

Definity contrast echocardiography showing apical wall thickening.
